# New York Letter

**Published:** 1891-12

**Authors:** 


### NEW YORK LETTER.

NEW YORK, Nov. 18th, 1891.

Dr. Beverly Robinson of this city, read at the section of practice at the New York Academy a very able, interesting and instructive paper on "The use of Creosote in the Treatment of Pulmonary Phthisis." He commenced by saying that he considered creosote the very best remedy we have for consumption, and its effects are very noticeable and merit recognition. The general symptoms of the disease are all greatly benefited by the use of this drug, the cough is diminished in frequency and severity, and even in the advanced stages, where cavities are present, the cough is ameliorated after some months of continued treatment. The expectoration is diminished in quantity and changed in quality, and after a little while it disappears altogether if the patient is still in the first stage of the disease. Nutrition is notably aided and this is shown by the increase in weight and the greater strength and activity. All the internal viscera, the lung, liver and bowels are functionally put in a better condition, the digestive processes are strengthened and the bowels are regulated. Night sweats often disappear during this treatment, and the temperature record is favorably influenced. In regard to the presence of the bacilli in the sputum; in some instances the expectoration was arrested and there were no bacilli in the sputa. The moist rales, limited to the apices, become fewer and have occasionally disappeared altogether. When cavities, where present, at the apices, the area of tissue contracted in a marked degree, and all the physical evidences of local disease were somewhat more satisfactory.

The contra-indications to the use of creosote are few and are easily obviated by a little judgment. Occasionally the stomach becomes intolerant after the administration of large quantities of the drug, and the way to correct this is to diminish the dose or interrupt its use for a little time. If diarrhoea ensue as a result of the use of creosote, the same rule holds good, or an appropriate dose of opium may be given in conjunction with this remedy.

In regard to the action of creosote on the kidneys, Dr.

Robinson has found after careful watching and repeated analyses, that creosote does not irritate these organs, except where large and repeated doses of the drug are taken. Should the patient manifest symptoms of haemoptysis, he considers it prudent to interrupt the use of creosote while the patient has haemoptosis or exhibits a tendency to it.

A large proportion of the so-called creosote dispensed in drugstores is nothing more or less than pure carbolic acid, and it is important to employ the creosote obtained from the beechwood and use no other.

Concerning the dose to be taken, he generally gives at the commencement from a half to one minim, and increases this amount every two or three days until the patient is able to take thirty drops, which amount may be reached in the space of nine or ten days. Creosote is the drug now employed for the treatment of phthisis in all of the large hospitals of New York, and he has met no one of the visiting physicians of these hospitals who has not confidence in its use.

Frequent and long continued inhalations of creosote he considers to be of great service when given in conjunction with its internal use. The larynx is at times very favorably modified by these inhalations, the mucous membrane becoming normal in color and the oedema and ulceration being greatly improved. Creosote when given in this way must be combined with alcohol in the proportion of one part of the former to eight of the latter. A perforated zinc plate in the form of inhaler is used and when worn for a certain period of time it affords great comfort and relief to the patient. About fifteen or twenty drops of the drug ought to be poured into the sponge of the inhaler.

Dr. Robinson gives in this paper the results of his own treatment of phthisical patients in St. Luke's and other hospitals of this city covering a pericd of a number of years and says that he considers creoscte in the first and second stages of phthisis a more efficient and certain remedy than any he has up to the present time been called upon to try.

Dr. W. G. Thompson said he had been using this drug for the last five years in increasing amount in the treatment of phthisis and he considered it important to saturate the patient with creosote as rapidly as possible. To this end he has been using the inhaler in conjunction with internal medication, and he has seen very great benefit accrue from the combined use. The most striking effect, he noticed were on the general nutrition, appetite and strength of the patient. It has not been his experience that the use had any bad effect on the kidneys, and he has seen no toxic effect result from the administration of this drug up to thirty minims, other than gastric disturbances which are liable to be produced by any remedy. For some years he has been treating phthisical patients with creosote alone, and he has found it the most efficient drug for the treatment of ordinary cases of phthisis in the first and second stages.

Dr. B. F. Curtis, visiting surgeon to the Presbyterian Hospital, recently operated upon a patient for osteomyelitis of the os-calcis. After the patient had been placed under ether in the operating room of the hospital and the parts rendered thoroughly aseptic, an incision was made down to the bone and the interior scraped thoroughly with a blunt curette; leaving a cavity of about one and a half inches in depth and of the same width. After it had been thoroughly scraped free of all granulations, decalcified chips of bone were then placed in the cavity inlayers, iodoform being dusted between each successive layer. After the cavity had been filled up in this way, the wound was then approximated by catgut sutures and rubber tissue placed over it so as to prevent the gauze from drying up the secretions too rapidly. Over this was laid a gauze dressing. This dressing will be permitted to remain in situ until the wound is healed.

Dr. W. Gill Wylie in a recent clinical lecture on fibroids of the uterus at the New York Polyclinic, spoke as follows: "Now, one word about fibroids. Some of you may think that the hemorrhage in fibroids comes direct from the tumor. That is a mistake A fibroid tumor of the uterus enlarges the blood vessels, and in that way influences the hemorrhage. If a woman has a fibroid and a hemorrhage, you can take it for an absolute certainty that she has also fungoid growths of the uterus. So in the case of fibroids where you have also a hemorrhage, if you can curette the uterus you will invariably stop the hemorrhage. These fibroids have a curious effect on the menopause.

If a woman of forty-four or five years of age has a fibroid, it will not do to say, wait for the menopause, for if it be a large fibroid it prolongs menstruation to the age of fifty-five or sixty. Furthermore, if the tumor be permitted to remain for that space of time, it is very apt to break down and give rise to the development of a peculiar form of sepsis.

